# Absence of toll-like receptor 9 Pro99Leu polymorphism in cervical cancer

**DOI:** 10.12688/f1000research.14840.2

**Published:** 2018-08-30

**Authors:** Alex Chauhan, Nilesh Pandey, Nitin Raithatha, Purvi Patel, Ajesh Desai, Neeraj Jain

**Affiliations:** 1 P D Patel Institute of Applied Sciences, Charotar University of Science and Technology (CHARUSAT), Changa, India; 2 Department of Obstetrics and Gynaecology, Pramukh Swami Medical College, Shree Krishna Hospital, Karamsad, India; 3 Department of Obstetrics and Gynaecology, Sir Sayajirao General Hospital and Medical College Baroda, Vadodara, India; 4 Department of Obstetrics and Gynaecology, GMERS Medical College and Hospital, Ahmedabad, India

**Keywords:** Cervical cancer, TLR9, Polymorphism, Genotypic frequency, Susceptibility

## Abstract

**Background:** Toll-like receptor 9 (TLR9) plays a key role in the elimination of viral pathogens by recognising their CpG DNA. Polymorphisms in the *TLR9* gene may influence their recognition and subsequent elimination. Therefore, the present study was designed to elucidate the role of a rare unexplored *TLR9* gene polymorphism C296T/ Pro99Leu (rs5743844) in cervical cancer susceptibility among Indian women.

**Methods: **The genotyping of *TLR9* Pro99Leu polymorphism in 110 cervical cancer patients and 141 healthy controls was performed by polymerase chain reaction and restriction fragment length polymorphism (PCR-RFLP).

**Results: **The genotype frequency detected in both cervical cancer and control populations was 1.0 (CC), 0.0 (CT) and 0.0 (TT); while the allele frequency was found to be 1.0 (C) and 0.0 (T).

**Conclusions: **The present study demonstrates no involvement of *TLR9* C296T/ Pro99Leu polymorphism in cervical cancer susceptibility and supports minor allele frequency (MAF) (0.0002) status of the same as no nucleotide variation was detected in any of the study subjects.

## Introduction

Cervical cancer is the fourth-most common cancer among women globally and second leading cause of cancer-related deaths in Indian women ^1^. Although persistent infection of high-risk human papillomavirus (hrHPV) is considered as the chief causative agent of cervical cancer, variations in host genetic make-up does influence the risk of acquiring HPV infection, and susceptibility to cervical carcinogenesis ^2– 4^. In this context, variations in Toll-like receptor ( *TLR*) genes, that play a crucial role in activating immune response by identifying pathogen-associated molecular patterns (PAMPs), have drawn significant attention, as single nucleotide polymorphisms (SNPs) in *TLR* genes have been shown to alter susceptibility to many infections and human diseases including cancer ^5– 8^.

Ten functional *TLR* genes are known in humans, the products of which recognizes specific PAMPs ^9^. TLR1, TLR2, TLR4, TLR5 and TLR6 which are located on the cell surface of immune cells are known to recognize triacyl lipopeptides, peptidoglycans, lipopolysaccharides, flagellin and diacyl lipopeptides respectively that belong to bacterial cell wall or virus particles ^10, 11^. On the other hand TLR3, TLR7, TLR8 and TLR9 are located in the endosomal compartments, wherein TLR3 recognizes single as well as double stranded viral RNA, TLR7 recognizes ssRNA from viruses while the TLR9 gene product recognizes bacterial and viral DNA motifs including HPV16 CpG motifs ^5, 10– 12^. Frequently analysed *TLR9* SNPs G2848A and −1486 T/C have been suggested to alter cervical cancer susceptibility ^13– 16^, but no report is available elucidating the role of *TLR9* Pro99Leu polymorphism in cancer. Although *TLR9* Pro99Leu is a rare population SNP with a global minor allele frequency (MAF) of 0.0002 as reported in the single nucleotide polymorphism database (dbSNP), *in-vitro* analysis has revealed its significant role in DNA ligand hyporesponsiveness ^17^. Considering the fact that cervical cancer is largely caused by hrHPV infection and TLR9 has the ability to respond to viral DNA, while other TLRs do not, the present study was designed to elucidate the association of the *TLR9* Pro99Leu polymorphism with cervical cancer.

## Methods

### Biological specimens

Biopsies from 110 cervical cancer patients and cervical smears from 141 healthy volunteers were collected from Shree Krishna Hospital, Anand; Sir Sayajirao General Hospital, Vadodara; and GMERS Hospital, Ahmedabad, India. The samples were collected from 2012 to 2017. The cancer biopsies and healthy cervical smears were histopathologically and cytologically confirmed. The clinical staging of cervical cancer samples was done as per The International Federation of Gynecology and Obstetrics (FIGO) guidelines.

### DNA isolation and genetic analysis

DNA was isolated from cervical cancer biopsies and cervical smears by standard phenol-chloroform extraction method ^18^. In the case of a low number of cervical cells, a spin-column based DNA isolation kit (Macherey-Nagel, Germany; Cat# 740952.50) was utilized as per manufacturer’s instructions. The quality and quantity of DNA was determined using ethidium bromide-stained 1% agarose gel on GelDoc system (BioRad, USA) as well as a NanoDrop 2000 (Thermofisher, USA). The *TLR9* Pro99Leu polymorphism was detected using polymerase chain reaction and restriction fragment length polymorphism (PCR-RFLP) method as described by Kubarenko *et al*. ^17^ Briefly, a 25µl PCR mix contained 0.1µM each of forward and reverse primer (Imperial Life Sciences, India), 0.1mM dNTP mix (Invitrogen, USA; Cat# 18427088), 2.5mM MgCl _2_ (Vivantis, USA; Cat# RB0204), 1 unit Taq DNA polymerase (Kapabiosystems, USA; Cat# KK1015) and 100 to 150ng genomic DNA. The PCR was run on an MJ Mini thermal cycler (BioRad, USA).

Upon confirmation of 337 bp PCR product on 2% ethidium bromide-stained agarose gel, 10µl PCR product was digested with *BslI* restriction enzyme (New England Biolabs, USA; Cat# R0555S) at 55°C overnight, separated on 12% polyacrylamide gel and analysed on a GelDoc system (BioRad, USA) for genotype identification. The details of PCR conditions and parameters for genotype consideration are mentioned in Table 1 and Table 2 respectively. To confirm the PCR-RFLP results, we performed Sanger sequencing of five randomly selected cervical cancer as well as control samples. All the sequencing reactions were performed on 3730 *xl* DNA Analyzer (Applied Biosystems, USA) using BigDye™ Terminator v3.1 kit (Applied Biosystems, USA; Cat# 4337454) as per manufacturer’s instructions. The 10µl sequencing reaction was comprised of 7.0µl BigDye™ Terminator v3.1 Ready Reaction Mix, 10pmol forward primer and 50ng PCR product. The sequencing results were analyzed on Sequencing Analysis Software version 5.3.1 (Applied Biosystems, USA).

**Table 1.** Details of *TLR9* C296T/ Pro99Leu specific PCR.

Primer Sequence (5′ – 3′)	Thermal Profile	PCR Product	Visualized on
FP: GGATGTTGGTATGGCTGAGG RP: AACTGCAACTGGCTGTTCCT	(95°C – 5′) 1 (95°C – 45″, 56°C – 1′, 72°C – 30″) 35 (72°C – 10′) 1	337 bp	2% Agarose Gel

Abbreviations: *TLR9, Toll-like receptor 9*; FP, forward Primer; RP, Reverse Primer; PCR, Polymerase Chain Reaction; bp, base pairs

**Table 2.** Parameters for genotypes consideration of *TLR9* C296T/ Pro99Leu polymorphism.

Enzyme	Digested Products (bp)	Genotype
***BslI***	166 + 136 + 35	CC (Pro/Pro)
	201 + 166 + 136 + 35	CT (Pro/Leu)
	201 + 136	TT (Leu/Leu)

### Statistical analysis

Statistical analysis was performed on GraphPad Prism version 5.00 for Windows (GraphPad Software, USA). Age of patients and controls were compared using two-sided Student's t-test. Due to the presence of single genotype across all the samples no additional statistical association was performed. The power of the study was calculated using Online Sample Size Estimator.

## Results

### Demographic and clinical characteristics

The average age of cervical cancer patients (52.43±11.78 years) and controls (51.8±11.35 years) was comparable without any statistically significant difference (p=0.668). Histopathologic analysis revealed all the cervical cancer cases to be of squamous cell carcinoma type. According to FIGO analysis, 9 (8.2%), 39 (35.5%), 55 (50%) and 7 (6.3%) patients belonged to Stage I, II, III and IV respectively. In the absence of polymorphic allele in the present population we calculated the power of the study based on the global minor allele frequency (0.0002) which was found to be 3.6%.

### *TLR9* Pro99Leu polymorphism

**Figure 1.**
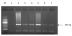
Representative PCR picture showing amplification of *TLR9* gene segment for C296T/ Pro99Leu gene polymorphism on ethidium bromide-stained 2% agarose gel. Lane M is 100 bp molecular marker (Takara, Japan; Cat# RR820A), Lane 1 is negative control and Lanes 2–7 are tumor DNA showing PCR products of 337 bp. (Abbreviations: PCR, Polymerase Chain Reaction; *TLR9, Toll-like receptor 9;* bp, base pair).

**Figure 2.**
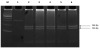
Representative PAGE picture of RFLP results for *TLR9* C296T/ Pro99Leu polymorphism on 12% polyacrylamide gel. Lane M is 100 bp molecular marker, Lane 1 is undigested PCR product and Lanes 2 to 6 are showing digested PCR products of 166 bp and 136 bp (35 bp band is not visible) by *BslI* enzyme representing CC genotype. (Abbreviations: PAGE, Polyacrylamide Gel Electrophoresis; RFLP, Restriction Fragment Length Polymorphism).

**Figure 3.**
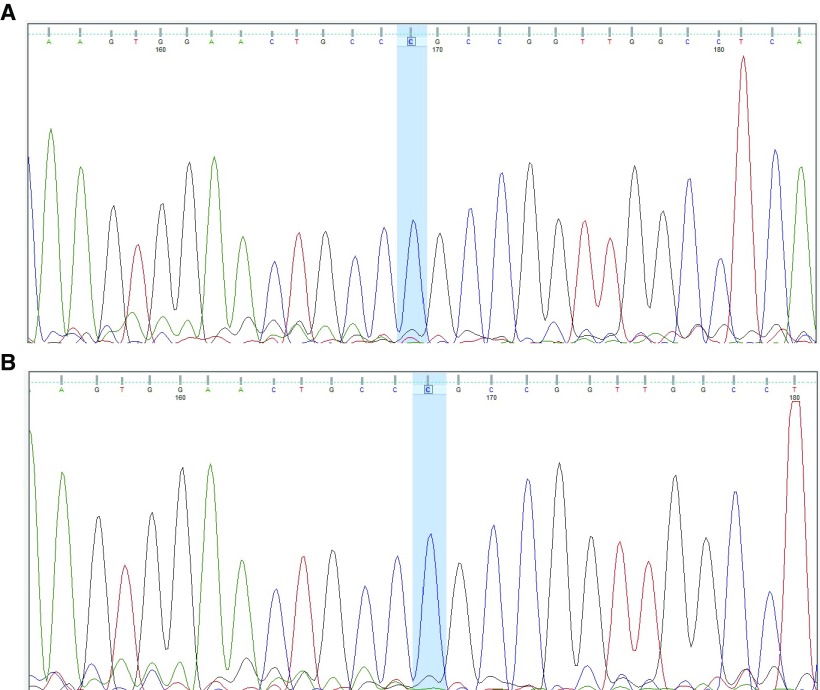
Sanger sequence electropherogram of ( **A**) a healthy individual and ( **B**) patient showing single peak (highlighted) of C allele of *TLR9* C296T/ Pro99Leu SNP representing CC genotype. (Abbreviations: SNP, Single Nucleotide Polymorphism).

PCR amplification revealed the presence of a single intact band of 337 bp ( Figure 1; Dataset 1 ^19^). A single genotype CC (Pro/Pro) was detected across all the sample types ( Table 3; Dataset 2 ^20^) which was evident by the presence of 166 bp, 136 bp and 35 bp DNA bands after RFLP assay ( Figure 2; Dataset 3 ^21^). Sanger sequencing of the randomly selected PCR products corroborated with RFLP results ( Figure 3; Dataset 4 ^22^).

**Table 3.** Genotype and allele frequencies of *TLR9* C296T/ Pro99Leu polymorphism in cervical cancer patients and healthy controls.

Genotype	Cervical Cancer *n* (%)	Controls *n* (%)
**CC**	110 (100.0)	141 (100.0)
**CT**	0 (0.0)	0 (0.0)
**TT**	0 (0.0)	0 (0.0)
**Allele**		
**C**	220 (100.0)	282 (100.0)
**T**	0 (0.0)	0 (0.0)

Raw agarose gel images of PCR amplification of *TLR9* gene segment for C296T/ Pro99Leu polymorphism from 50 samples consisting of 26 controls and 24 cervical cancer cases Figure 1 is a representative picture of the same. Click here for additional data file. Copyright: © 2018 Chauhan A et al. 2018 Data associated with the article are available under the terms of the Creative Commons Zero "No rights reserved" data waiver (CC0 1.0 Public domain dedication).

Age, clinical stage and *TLR9* genotype status among cervical cancer patients as well as age and *TLR9* genotype status among controls Click here for additional data file. Copyright: © 2018 Chauhan A et al. 2018 Data associated with the article are available under the terms of the Creative Commons Zero "No rights reserved" data waiver (CC0 1.0 Public domain dedication).

Raw polyacrylamide gel electrophoresis images of 27 controls and 24 cervical cancer PCR amplified products that underwent restriction fragment length polymorphism (RFLP) analysis Figure 2 is a representative picture of the same. Click here for additional data file. Copyright: © 2018 Chauhan A et al. 2018 Data associated with the article are available under the terms of the Creative Commons Zero "No rights reserved" data waiver (CC0 1.0 Public domain dedication).

Nucleotide sequences spanning *TLR9* gene segment for C296T single nucleotide polymorphism, obtained after performing Sanger sequencing on five samples each of cervical cancer and healthy controls The sequencing results confirm the restriction fragment length polymorphism (RFLP) analysis that represents single genotype CC among all the study subjects. Figure 3A and B are representative electropherograms of the *TLR9* C296T CC genotype as evident by the presence of single peak of C allele. Click here for additional data file. Copyright: © 2018 Chauhan A et al. 2018 Data associated with the article are available under the terms of the Creative Commons Zero "No rights reserved" data waiver (CC0 1.0 Public domain dedication).

## Discussion

Although hrHPV infection is the primary etiological agent of cervical carcinogenesis, the role of host genetic factors, especially those associated with body immunity such as TLRs, cannot be ignored. TLR9 which recognizes CpG DNA motifs from bacteria and viruses has also been reported to recognize HPV16 CpG DNA ^12^. Moreover, variations in the *TLR9* gene has found associations with various diseases including cancer. *TLR9* SNPs −1486 T/C and G2848A have been found to be contradictorily associated with cervical cancer risk. In Polish and Mexican populations both *TLR9* −1486 T/C and G2848A polymorphisms were suggested to be risk factors for cervical carcinogenesis ^13, 15^. In two independent studies on Chinese population, a positive association with *TLR9* G2848A SNP was detected ^23, 24^ but no involvement of *TLR9* −1486 T/C was found ^24^, however, the other study suggested −1486 T/C was not a contributory factor to cervical carcinogenesis ^14^. From India, a single report on North Indian patients revealed a marginal role of *TLR9* G2848A polymorphism with cervical cancer risk ^16^.

To date, no report is available on the rare *TLR9* Pro99Leu polymorphism in cancer, which has been shown to be associated with DNA ligand hyporesponsiveness in HeLa cell lines ^17^. Considering the fact that cervical cancer is majorly caused by hrHPV infection and the *TLR9* Pro99Leu polymorphism is associated with DNA ligand hyporesponsiveness, the present study investigated, for the first time, the role of the *TLR9* Pro99Leu polymorphism in cervical cancer susceptibility. This is also the first report to study this polymorphism in any of the cancer types globally. Our results revealed the presence of a single genotype CC (Pro/Pro) among cases and controls, demonstrating no significance of the Pro99Leu polymorphism to cervical cancer susceptibility. Similarly, no association with high-risk HPV infection, that was detected in almost 70% of the patients, was found (details of HPV infection to be published elsewhere). A complete absence of Pro99Leu in our study population corroborates with the report of Lee and group (2006) where neither controls nor lung tuberculosis and sarcoidosis patients had the *TLR9* Pro99Leu polymorphism ^25^. Similarly, the Pro99Leu polymorphism was not detected among healthy Caucasians as well as pneumococcal disease, bacteraemia, and leprosy patients ^17^. Moreover, according to dbSNP, the global MAF of this polymorphism is 0.0002, and our results, albeit on a smaller cohort, do solicit its rare polymorphism status. Therefore, owing to sample size constraint, it would be inappropriate to draw a direct conclusion of the effect of above-said polymorphism on the susceptibility to cervical cancer. As the power of study calculated based on the global MAF was very low (3.6%), and to achieve a power of study of 80%, approximately 40000 samples will be required to analyze. Finally, under present scenario, a direct role of this SNP in cancer, as well as other diseases, seems a remote possibility. Nonetheless, a comprehensive analysis of a larger cohort covering a varied ethnic population globally is suggested to comprehend its role in microbial infection and/or disease susceptibility including cancer.

## Conclusion

The preliminary data obtained from the present study does not suggest a role for the *TLR9* Pro99Leu polymorphism in cervical cancer susceptibility. However, analysis on a larger cohort worldwide may provide more insights into the frequency distribution of Pro99Leu polymorphism and reveal its influential role in various human diseases including cancer. 

## Data availability

The data referenced by this article are under copyright with the following copyright statement: Copyright: © 2018 Chauhan A et al.

Data associated with the article are available under the terms of the Creative Commons Zero "No rights reserved" data waiver (CC0 1.0 Public domain dedication). 

Dataset 1. Raw agarose gel images of PCR amplification of *TLR9* gene segment for C296T/ Pro99Leu polymorphism from 50 samples consisting of 26 controls and 24 cervical cancer cases. Figure 1 is a representative picture of the same. 10.5256/f1000research.14840.d203405 ^19^

Dataset 2. Age, clinical stage and *TLR9* genotype status among cervical cancer patients as well as age and *TLR9* genotype status among controls. 10.5256/f1000research.14840.d203406 ^20^

Dataset 3. Raw polyacrylamide gel electrophoresis images of 27 controls and 24 cervical cancer PCR amplified products that underwent restriction fragment length polymorphism (RFLP) analysis. Figure 2 is a representative picture of the same. 10.5256/f1000research.14840.d203407 ^21^

Dataset 4. Nucleotide sequences spanning *TLR9* gene segment for C296T single nucleotide polymorphism, obtained after performing Sanger sequencing on five samples each of cervical cancer and healthy controls. The sequencing results confirm the restriction fragment length polymorphism (RFLP) analysis that represents single genotype CC among all the study subjects. Figure 3 A and B are representative electropherograms of the *TLR9* C296T CC genotype as evident by the presence of single peak of C allele. 10.5256/f1000research.14840.d203408 ^22^

## Ethical considerations

The research was carried out following due approval from ethics committee of all the participating institutes. Subjects were verbally informed and explained about the study, and were provided with an information sheet. Written informed consent was obtained from the subjects who agreed to enrol in the present study. Personal information of all the study subjects was kept confidential.
