# Supplementary material for: Absence of toll-like receptor 9 Pro99Leu polymorphism in cervical cancer
Source: F1000Res. 2018 Aug 30;7:606. Originally published 2018 May 17. [Version 2] doi: 10.12688/f1000research.14840.2 (PMC6171715; doi:10.12688/f1000research.14840.2)
Supplement: Peer review history

# Peer review: Referee response for version 2

**DOI:** 10.5256/f1000research.17000.r39042

**Published:** 2018-10-03

**Authors:** Gopeshwar Narayan

**Affiliations:** 1 Department of Molecular and Human Genetics, Banaras Hindu University, Varanasi, India

The authors have addressed the issues raised in the previous version.

I have read this submission. I believe that I have an appropriate level of expertise to confirm that it is of an acceptable scientific standard.

# Peer review: Referee response for version 2

**DOI:** 10.5256/f1000research.17000.r37741

**Published:** 2018-09-13

**Authors:** Balraj Mittal

**Affiliations:** 1 Department of Biotechnology, Babasaheb Bhimrao Ambedkar University, Lucknow, India

I had already mentioned in my original review that it is useless to look for association of a monophonic allele with a disease and study has zero statistical power. The only conclusion from the present study is that *TLR9* C296T/ Pro99Leu polymorphism appears to be monomorphic in Gujrati Indians. However, it again suffers from low sample size. The person designing such study has to understand the basic tenets of genetic epidemiology. Therefore, I do not feel that the manuscript deserves indexing.

I have read this submission. I believe that I have an appropriate level of expertise to state that I do not consider it to be of an acceptable scientific standard, for reasons outlined above.

# Peer review: Referee response for version 2

**DOI:** 10.5256/f1000research.17000.r37740

**Published:** 2018-09-04

**Authors:** Bhudev C. Das

**Affiliations:** 1 Dr. B. R. Ambedkar Center for Biomedical Research, University of Delhi, New Delhi, India

I have gone through the revised manuscript entitled "Absence of toll-like receptor 9 Pro99Leu polymorphism in cervical cancer" submitted by Alex Chauhan et al. The authors have revised the manuscript satisfactorily and answered all the queries therein. The English corrections that I asked for has been incorporated in the revised manuscript and I now recommend final acceptance of the manuscript for indexing of the article.

I have read this submission. I believe that I have an appropriate level of expertise to confirm that it is of an acceptable scientific standard.

# Peer review: Referee response for version 1

**DOI:** 10.5256/f1000research.16153.r34120

**Published:** 2018-08-02

**Authors:** Bhudev C. Das

**Affiliations:** 1 Dr. B. R. Ambedkar Center for Biomedical Research, University of Delhi, New Delhi, India

I have gone through the manuscript entitled “Absence of toll-like receptor 9 Pro99Leu polymorphism in cervical cancer” submitted by Alex Chauhan et al. for its publication in F1000Research. The authors have studied the polymorphisms of Toll-like receptor 9 (TLR9) Pro99Leu polymorphism in cervical cancer to examine its role in cervical carcinoma in elimination of viral pathogens though recognition of CpG islands. It is suggested that polymorphism of TLR9 gene may influence the recognitions of the above DNA sequence leading to elimination of infection. The authors have used as many as 110 cervical cancer samples and normal cervical smears from 141 healthy controls and employed PCR-RFLP and sequencing methods for detection of genotype variation. The authors could not find any variation in genotypes of TLR9 hence authors concluded that the specific polymorphism C296T/Pro99Leu has no role in cervical cancer.

 This is a very clean and straight forward study which could not find any link between TLR9 polymorphism and cervical cancer. The data and the figures presented including PCR and PCR-RFLP and sequencing pictures are excellent and convincing. However, there are few points which need to be clarified/corrected before the manuscript is accepted and indexed. My comments are as follows:

1. The whole study is based on only one TLR9 gene polymorphism. Authors need to very clearly justify their choice of TLR9, and not other TLRs in Introduction as well as in the Discussion.
2. It is well established that the causative agent for cervical cancer is due to infection of specific types of high risk Human papillomaviruses. Any study on cervical cancer demands for an obvious correlation/association with HPV status of the cervical cancer. If at all no HPV analysis has been done, the authors must discuss this in the Discussion.
3. There are several English and grammatical errors throughout the manuscript. Authors need to carefully re-read the manuscript and correct the manuscript. Few obvious errors are indicated here:- 
  i) Methods: 3 ^rd^ line: reaction **and **restriction fragment length
  ii)Conclusion: 1 ^st^ line – the present study demonstrates no involvement – (delete ‘results’), 3 ^rd^ line – delete ‘worldwide’ and in 4 ^th^ line – delete ‘participants’, replace it with ‘subjects 
  iii)Table 1 last column below Visualized on: write 2% Agarose gel.
4. Data set 3: delete ‘unedited’.

In summary, the manuscript may be accepted for indexing after the authors made the minor revision of the manuscript as suggested above.

I have read this submission. I believe that I have an appropriate level of expertise to confirm that it is of an acceptable scientific standard, however I have significant reservations, as outlined above.

# Response

**Published:** 2018-08-20

**Authors:** Alex Chauhan

**Affiliations:** P D Patel Institute of Applied Sciences, India

Dear Prof. B. C. Das,

 We thank you for approving our manuscript and appreciate your valuable suggestions. Please find below the response towards your reservations:

**Reservation 1:** The whole study is based on only one *TLR9 *gene polymorphism. Authors need to very clearly justify their choice of TLR9, and not other TLRs in Introduction as well as in the Discussion.

**Response:** The choice of TLR9 is based on the fact that it recognized HPV16 DNA which is a main causative agent of cervical cancer. As suggested this has been included in the introduction as well as discussion. 

**Reservation 2: **It is well established that the causative agent for cervical cancer is due to infection of specific types of high risk Human papillomaviruses. Any study on cervical cancer demands for an obvious correlation/association with HPV status of the cervical cancer. If at all no HPV analysis has been done, the authors must discuss this in the Discussion.

**Response 2:** We do agree that cervical cancer is mainly caused by the infection of hrHPVs. We found approximately 70% of our cases with HPV positivity, that has been incorporated in the discussion. The details of HPV infection will be published elsewhere.

**Reservation 3:** There are several English and grammatical errors throughout the manuscript. Authors need to carefully re-read the manuscript and correct the manuscript. Few obvious errors are indicated here:- 

 i) Methods: 3rd line: reaction and restriction fragment length

 ii)Conclusion: 1st line – the present study demonstrates no involvement – (delete ‘results’), 3rd line – delete ‘worldwide’ and in 4th line – delete ‘participants’, replace it with ‘subjects 

 iii)Table 1 last column below Visualized on: write 2% Agarose gel.

**Response 3:** The above mentioned suggestions have been incorporated.

**Reservation 4:** Data set 3: delete ‘unedited’.

**Response 4: **Deleted.

# Peer review: Referee response for version 1

**DOI:** 10.5256/f1000research.16153.r35718

**Published:** 2018-07-23

**Authors:** Balraj Mittal

**Affiliations:** 1 Department of Biotechnology, Babasaheb Bhimrao Ambedkar University, Lucknow, India

The basic assumption of genetic epidemiology is that two alleles of a gene are present in the population and one of the allele (usually minor) has altered frequency in cases and controls. However, if a gene is mono-morphic as is the case here for TLR9C296T, then it is non-informative and there is no point to look for its association.

 Therefore, the very basis of study is wrong, even though it has been carried out technically correct.

 I therefore feel that this publication will not anything the present knowledge.

I have read this submission. I believe that I have an appropriate level of expertise to state that I do not consider it to be of an acceptable scientific standard, for reasons outlined above.

# Peer review: Referee response for version 1

**DOI:** 10.5256/f1000research.16153.r34771

**Published:** 2018-06-25

**Authors:** Gopeshwar Narayan

**Affiliations:** 1 Department of Molecular and Human Genetics, Banaras Hindu University, Varanasi, India

With the global minor allele frequency of 0.0002, the studied sample size is too small. It is interesting to observe that there is only one genotype (homozygous wild type) present in the studied cohort. The power of the study should be mentioned by the authors. The conclusion drawn from the limited data set may not reflect the real situation. I suggest the authors to first calculate the number of samples required for the study on the basis of the frequency of minor/major alleles to achieve about 80% power of study and increase the sample size accordingly.

I have read this submission. I believe that I have an appropriate level of expertise to confirm that it is of an acceptable scientific standard, however I have significant reservations, as outlined above.

# Response

**Published:** 2018-06-29

**Authors:** Alex Chauhan

**Affiliations:** P D Patel Institute of Applied Sciences, India

Dear Dr. Gopeshwar Narayan, 

 We thank you for approving our manuscript and appreciate your valuable suggestions. Please find below the response towards your reservations:

**Reservation:** With the global minor allele frequency of 0.0002, the studied sample size is too small. It is interesting to observe that there is only one genotype (homozygous wild type) present in the studied cohort. The power of the study should be mentioned by the authors. The conclusion drawn from the limited data set may not reflect the real situation. I suggest the authors first to calculate the number of samples required for the study on the basis of the frequency of minor/major alleles to achieve about 80% power of study and increase the sample size accordingly.

**Response:** Due to the complete absence of minor allele, the power of present study cannot be calculated. However, considering the global minor allele frequency of 0.0002 of the SNP, we calculated the power of study using Online Sample Size Estimator which was found to be 3.6%. To achieve 80% power of study approximately 40000 cases and controls are required. It is presently not possible for us to collect and analyze such a larger sample size. Studies on Pro99Leu polymorphism with similar sample size and results have also been reported by Kubarenko et al., 2010 and Lee et al., 2006, which have been cited in the article.
